# Mechanistic Insights into NDMA Adsorption onto Selected Pollutants and Their Removal via Direct Rapid Sand Filtration and After Enhanced Coagulation

**DOI:** 10.3390/molecules30102094

**Published:** 2025-05-08

**Authors:** Olubunmi M. Olukowi, Tian Tian, Xie Yan, Yuejun Zhang

**Affiliations:** School of Chemical Engineering, Nanjing University of Science and Technology, 200 Xiao Ling Wei, Nanjing 210094, China; olukowiolubunmi@njust.edu.cn (O.M.O.); tian980808@163.com (T.T.); xyanyaner@163.com (X.Y.)

**Keywords:** adsorption, simulated water, water pollutant parameters, NDMA removal mechanism, rapid sand filtration

## Abstract

N-nitroso dimethylamine (NDMA), a common nitrogen disinfection by-product and carcinogen, can be removed using rapid sand filtration (RSF) after coagulation; however, its removal mechanism has not been extensively studied. This study analyzed NDMA and the water pollutant parameter removal rate change tendency in the filtrates of simulated supernatants directly and after enhanced coagulation (EC) using composite PAC/PDMDAAC that mimics treated Yangtze River water separated into blank, single-component, and mixed multi-component (MMC) water systems containing NDMA and pollutants like diatomite (DTA), humic acid salt (HAs), dimethyl amine (DMA), and ammonium nitrate (NH_4_NO_3_). Meanwhile, a correlation analysis of removal rate changes and adsorption analysis using SEM (surface morphology), polar functional groups, and zeta potentials (surface charge) were performed to obtain mechanistic insights into NDMA removal via adsorption. The results revealed that removal rates gradually increased with an increasing volume of filtrates, and there were correlations for NDMA-HAs, NDMA-DMA, NDMA-DTA, and NDMA-NH_4_NO_3_. The highest NDMA removal rates in the blank system were 10.29% using RSF directly and 12.84% after enhanced coagulation, indicating improved efficiency with coagulation. However, single and mixed systems showed that NDMA removal rate changes were enhanced by water pollutants and coagulation functions. The NDMA removal mechanism was verified, and it was revealed that the level of NDMA adsorption on water pollutants varies based on microstructure, available polar functional groups, and surface charge interactions that are strengthened by coagulation functions for improving the affinity of NDMA and pollutants on the sand surface. These findings provide new insights into NDMA removal mechanisms via adsorption and highlight the role of water pollutants and enhanced coagulation in strengthening rapid sand filtration for NDMA removal.

## 1. Introduction

N-nitroso dimethylamine (NDMA), a nitrogenous disinfection by-product (N-DBP), is a widespread emerging pollutant in surface raw water reported to have mutagenic and carcinogenic properties [1]. NDMA is generated by the chemical oxidation of natural organic matter, dissolved organic nitrogen, and other NDMA precursors, mainly with chlorine or chloramine, as well as other alternative disinfectants (e.g., chlorine dioxide, ozone) [2,3,4]. Its high solubility and neutral charge make it difficult to remove, yet even low concentrations (0.7 ng/L) pose significant cancer risks [5,6].

Conventional treatment processes remove suspended particles, natural organic matter (NOM), and dissolved nitrogen pollutants, but NDMA removal remains a challenge [7,8,9,10,11]. Although many technologies have been reported on their use in NDMA removal in different water systems with different removal rates, such as reverse osmosis (85–95%), ultra-violet technology (80–90%), granular activated carbon (60–80%), and advance oxidation processes (90–95%), they are impractical for large-scale surface raw water treatment [12,13,14]. However, filtration after coagulation, widely used for surface water treatment, has shown promise in NDMA removal, particularly with enhanced coagulation using composite coagulants like poly aluminum-poly-dimethyldiallylammonium chloride (PAC/PDMDAAC) [15,16,17,18]. Adsorption onto sand during filtration is influenced by microstructure, surface charge, and molecular size, with larger particles removed by straining and smaller pollutants via adsorption [19,20,21]. However, limited studies have examined NDMA adsorption and removal in real water systems. Previous research, conducted in aqueous solutions with high NDMA concentrations with different media, reported low removal rates (<10%), leading to assumptions about the inefficacy of filtration. More studies are needed to assess NDMA removal in practical surface water treatment settings [22,23,24,25].

Our research group extensively studied NDMA and water pollution parameter removal using sand and activated carbon filtration after the conventional and enhanced coagulation of micro-polluted surface raw water. Initial studies by Xi [26] (explored NDMA removal using composite coagulant PAC/PDMDAAC with controlled supernatant turbidity at 2.0 NTU, achieving removal rates of 39% and 34.6% with activated carbon and rapid sand filtration, respectively. Zhou [27] further examined the effectiveness of PAC/PDMDAAC in autumn Yangtze River water, comparing different coagulant compositions under controlled turbidity (1.0–1.5 NTU). The optimal composite coagulant (1.60 dL/g, 10:1 PAC to PDMDAAC) achieved a 95.01% NDMA removal rate, surpassing PAC only (78.07%). A subsequent study in summer 2022 confirmed these findings, with the best-performing coagulant (1.50 dL/g, 10:1) achieving 92.31% NDMA removal, compared to 90.51% with PAC alone. Across multiple studies, enhanced coagulation consistently achieved NDMA removal rates above 90%, highlighting its effectiveness in surface raw water treatment.

Beyond the high NDMA removal rates achieved, our research group observed correlations between NDMA and water quality parameter removal trends, inspiring preliminary assumptions about the NDMA removal mechanism. It was assumed that residual suspended particles in supernatants, acting as carriers for NDMA, were transported and attached to sand filters during filtration. Additionally, residual coagulants were assumed to modify surface charges, enhancing NDMA adsorption. A recent NDMA removal mechanism initiated by enhanced coagulation revealed that NDMA removal depends on pollutant microstructure and polar functional groups; detailed mechanistic insights remain unknown for coagulation coupled with filtration. The complexity of surface raw water composition has hindered the direct observation and quantitative analysis of NDMA removal mechanisms. The effects of residual water pollutants in the supernatant on the NDMA removal mechanism have not been studied to the best of our knowledge, and the correlations among them are not clear, particularly in real surface raw water. Until now, studies on NDMA removal mechanisms have been based on preliminary assumptions, with limited studies investigating the role of residual water pollutants and their correlations with NDMA removal, particularly in real-world conditions.

In this study, based on existing NDMA removal rate studies, NDMA removal mechanism assumptions based on rapid sand filtration, and the new recognition of the NDMA removal mechanism enabled by enhanced coagulation, a new method was designed to simulate supernatants directly and after the enhanced coagulation of simulated Yangtze River raw water containing both NDMA and water pollutants such as diatomite (DTA), humic acid salt (HAs), dimethylamine (DMA), and ammonium nitrate (NH_4_NO_3_). Subsequently, the two kinds of supernatants used for blank, various single-component, and mixed multi-component water systems were used for sand filtration to obtain the NDMA and water pollutant parameter removal rates. These were analyzed to obtain the correlation of their removal rates and change tendency, while zeta potential and SEM analysis before and after filtration were also performed. Moreover, adsorption analysis in terms of the surface morphology, polar functional groups, and surface charge of pollutants with sand filters was also conducted to obtain new insights into the NDMA removal mechanism via adsorption for the wider application of enhanced coagulation with rapid sand filtration technology for NDMA removal treatment and lay the foundation for further theoretical studies for multiple nitrosamine removal.

## 2. Results and Discussion

### 2.1. NDMA and Water Pollution Parameter Removal Regulations in Various Rapid Sand Filtration Water Systems

#### 2.1.1. NDMA Removal Regulations in Terms of Rapid Sand Filtration of Simulated Supernatants for Blank Water System

**Figure 1:**
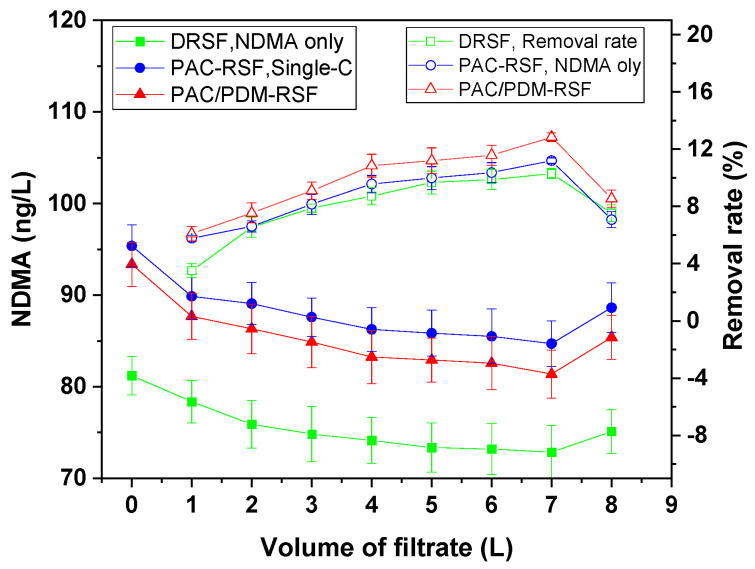
NDMA removal regulations in rapid sand filtration of supernatant directly and after enhanced coagulation for blank system.

Based on an experimental design, the rapid sand filtration of simulated supernatants was conducted both directly and after enhanced coagulation for blank water systems containing NDMA only. The resulting filtrate samples from the blank system were analyzed for their NDMA level, and the results are presented in Figure 1, which shows the residual NDMA removal rates at varying volumes of filtrate.

The NDMA removal tendencies of both the rapid sand filtration of the simulated supernatant directly (RSF-SSD) and the rapid sand filtration of the simulated supernatant after enhanced coagulation (RSF-SSEC) followed similar patterns when compared to conventional coagulation. The removal rates gradually increased with an increasing volume of filtrate, reaching the maximum after 7 L. At the highest removal point, RSF-SSD achieved a residual NDMA level of 71.89 ng/L from an initial level of 80 ng/L, corresponding to a removal rate of 10.14%. In contrast, for the supernatant treated with conventional coagulation using PAC and enhanced coagulation using the composite coagulant PAC/PDMDAAC, the residual NDMA concentrations were 84.70 ng/L (removal rate: 11.19%) and 81.38 ng/L (removal rate: 12.84%), respectively, from an initial concentration of 100 ng/L. The lower NDMA removal rate observed in RSF-SSD may be due to the stronger affinity of NDMA for water and its small molecular size (0.45 nm), which is relatively smaller than the sand pore size [5,23]. This is consistent with the low NDMA removal levels in aqueous solutions reported in the literature [22,23].

In contrast, RSF-SSEC using the composite PAC/PDMDAAC coagulant showed the highest NDMA removal efficiency, surpassing PAC-simulated water and RSF-SSD by 1.65% and 2.55%, respectively. These removal improvements are due to the enhanced coagulation effect, which facilitates adsorption on sand surfaces through residual polymer adsorption sites and a straining mechanism due to the large molecular size of PDMDAAC relative to sand pores. The highly positive charges and numerous adsorption sites in the residual PAC/PDMDAAC coagulant may have contributed to increased NDMA adsorption on sand surfaces. Furthermore, previous studies [28] have indicated that residual PAC/PDMDAAC acts as a filter aid due to its high molecular weight and charge density, further improving water quality parameter removal compared to PAC only. These findings highlight the importance of coagulation, especially enhanced coagulation, in improving NDMA removal efficiency via adsorption on sand, showing its advantages over direct filtration without coagulation.

#### 2.1.2. NDMA and Water Pollutant Parameter Removal Regulations in Terms of Rapid Sand Filtration of Simulated Supernatants for Various Single-Component Systems

**Figure 2:**
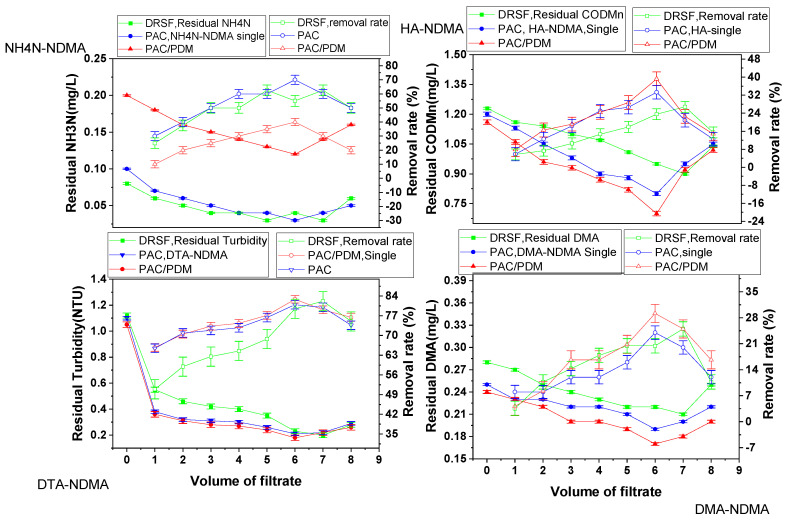
Water pollutant parameter removal regulations in rapid sand filtration of simulated supernatant after enhanced coagulation for various single-component systems.

**Figure 3:**
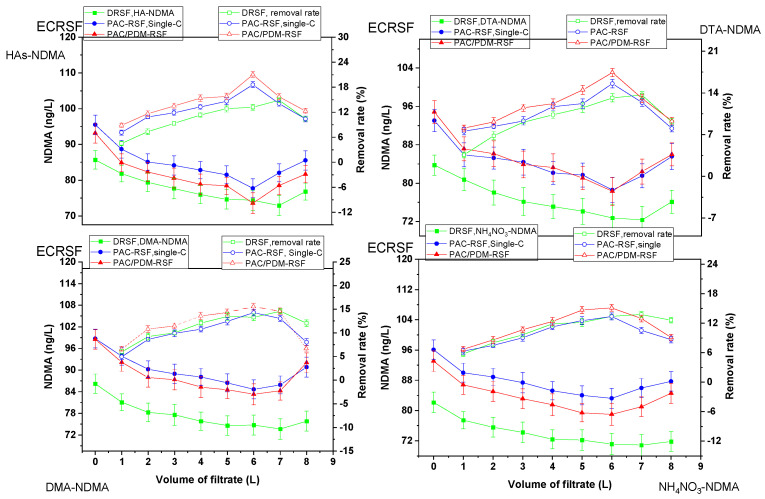
NDMA removal regulations in rapid sand filtration of simulated supernatant after enhanced coagulation for various single-component systems.

Based on an experimental design, the rapid sand filtration of simulated supernatants was conducted both directly (RSF-SSD) and after enhanced coagulation (RSF-SSEC) for various single-component systems, including DTA-NDMA, HAs-NDMA, DMA-NDMA, and NH_4_NO_3_-NDMA. The resulting filtrate samples were analyzed for NDMA and the corresponding water pollutant parameters, as well as zeta potentials. The results are presented in Figure 2 and Figure 3, which show the residual water pollutant parameter and NDMA removal change tendency in the RSF-SSD and RSF-SSEC systems. Additional details on RSF-SSD performance are provided in Figures S2 and S3 (Supplementary Material). Table 1 summarizes the statistical correlation between NDMA and water pollutant removal efficiencies in both single-component and multi-component systems, with further observational correlations available in Tables S1 and S2 (Supplementary Material).

On the one hand, the rapid sand filtration of the simulated supernatant directly (RSF-SSD) for single-component systems containing both NDMA and selected water pollutants was reported. The NDMA removal change tendency increased with filtrate volume, reaching a breakthrough point at 7 L, DTA-NDMA at 13.66%, HAs-NDMA at 14.90%, DMA-NDMA at 14.59%, and NH_4_NO_3_-NDMA at 13.78%, surpassing NDMA-only removal by 3.37–4.61%. The highest pollutant removal rates were 80.98% (turbidity), 26.82% (COD_Mn_), 25.00% (DMA), and 62.50% (NH_3_-N).

The order of NDMA adsorption on tested pollutants was HAs-NDMA > DMA-NDMA > NH_4_NO_3_-NDMA > DTA-NDMA > NDMA, suggesting that removal trends depend on pollutant microstructure, polar functional groups, and coagulation function. This confirms our previous NDMA removal mechanism assumption that NDMA removal via sand was strongly related to the adsorption of the removal of residual suspended particles with NDMA. For instance, humic acid salt has the highest NDMA removal rate due to its large molecular size with a well-distributed porous structure relative to sand pores; it has many functional groups with high polarity based on FTIR and SEM analysis, including a high polarity of attached groups as indicated by dipole moments such as carboxylic groups (1.6 D), a hydroxyl group (1.85 D), and an amino group (1.3 D) reported in the literature [29,30]. This result showed that residual diatomite, humic acid salt, DMA, and NH_4_NO_3_ contributed to strengthening the adsorption of NDMA onto the sand grain surface, leading to increased NDMA removal.

On the other hand, a comparison of the rapid sand filtration of the simulated supernatant after enhanced coagulation (RSF-SSEC) versus conventional coagulation (RSF-SSCC) was performed for various single-component systems, and the results are presented in Figure 2 and Figure 3 and Table 1. The results confirmed that enhanced coagulation using composite PAC/PDMDAAC significantly improved NDMA removal over conventional coagulation (PAC) and RSF-SSD. This is because PAC/PDMDAAC provided higher charge neutralization and adsorption sites, enhancing NDMA and pollutant removal [15,28].

The NDMA removal rate change tendency gradually increased with increasing volume for RSF-SSEC and RSF-SSCC, reaching their peak at 6 L (breakthrough volume) with NDMA-DTA at 17.42% and 15.54%; NDMA-HAs at 21.03% and 18.60%; NDMA-DMA at 15.45% and 14.28%; and NDMA-NH_4_NO_3_ at 15.12% and 13.37% for RSF-SSEC and RSF-SSCC, respectively. The NDMA removal rate in RSF-SSEC was 0.86–6.13% higher than that in RSF-SSD and 1.17–2.43% higher than that in RSF-SSCC, depending on the single-component system. These results indicate that the presence of water pollutants enhances NDMA adsorption both directly and indirectly through coagulation mechanisms, particularly PDMDAAC’s adsorption bridging effect.

The observed order of the NDMA removal rate tendency was HAs-NDMA > DTA-NDMA > DMA-NDMA > NH_4_NO_3_-NDMA > NDMA, suggesting that removal efficiency depends on pollutant microstructure, polar functional groups, and coagulation effects. The corresponding removal rates of water pollutants measured as turbidity (diatomite), COD_Mn_ (humic acid), NH_3_-N (ammonium nitrate), and DMA (dimethyl amine) were 82.86% and 80.98%; 39.23% and 33.30%; 31.25% and 23.30%; and 40.00% and 70.00% for RSF-SSEC and RSF-SSCC at a 6 L breakthrough volume. As expected, RSF-SSEC achieved the highest NDMA and pollutant removal rates compared to RSF-SSCC and RSF-SSD. These results confirm that NDMA adsorption onto sand is influenced by diatomite (turbidity), humic acid salt (organic matter), ammonium nitrate (NH_3_-N), and dimethyl amine (organic nitrogen), which interact with NDMA to varying degrees. Coagulation further enhances these interactions by modifying surface charges, functional groups, and polarity, ultimately increasing NDMA removal efficiency.

The shorter breakthrough volume (6 L) and the positive zeta potential of sand filtrates after coagulation indicate that residual water pollutants and coagulants enhanced NDMA adsorption by increasing the affinity of sand pores and surfaces. The improved NDMA removal efficiency in RSF-SSEC can be attributed to the presence of residual diatomite, humic acid salt, dimethyl amine, and ammonium nitrate, as well as the enhanced coagulation effects on surface charge interactions. Therefore, it can be concluded that the effects of coagulation, especially enhanced coagulation, on surface charges, in addition to the microstructure, available functional groups, and high polarity of the residual water pollutants after coagulation, strengthened the sand filtration process for the removal of NDMA via adsorption in a single-component system.

#### 2.1.3. NDMA and Water Pollution Parameter Removal Regulations in Rapid Sand Filtration of Simulated Supernatants for Mixed Multi-Component Systems

**Figure 4:**
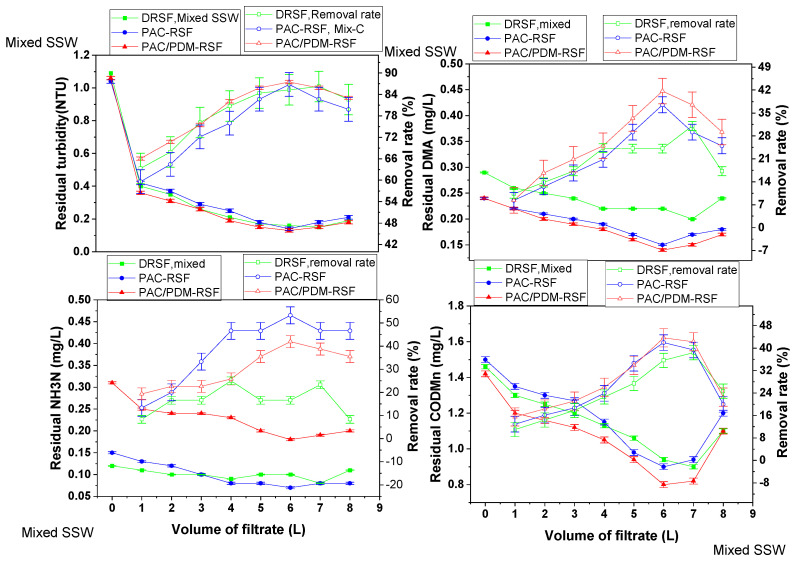
Water pollutant parameter regulations in rapid sand filtration of simulated supernatant after enhanced coagulation for mixed multi-component system.

**Figure 5:**
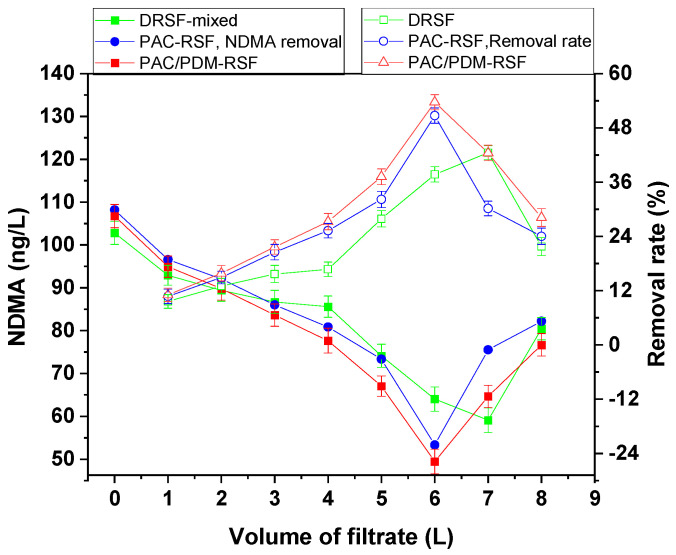
NDMA removal rate change tendency in rapid sand filtration of simulated supernatant after enhanced coagulation for mixed multi-component system.

Based on an experimental design, the rapid sand filtration of simulated supernatants was conducted under three conditions for mixed multi-component systems: direct filtration (RSF-SSD), filtration after enhanced coagulation (RSF-SSEC), and filtration after conventional coagulation (RSF-SSCC). The resulting filtrates were analyzed for NDMA and the corresponding water pollutant parameters to monitor their adsorption and removal. Figure 4 and Figure 5 show water pollutant parameters and NDMA removal regulations, while Figures S3 and S4 (Supplemental Materials) provide additional data. Table 1 shows the correlation found using a statistical method between NDMA and water pollution removal regulations for both single- and mixed multi-component systems, and the correlation obtained by direct observation is shown in Table S2. 

In the rapid sand filtration of the simulated supernatant directly (RSF-SSD) for mixed multi-component systems, it was observed that the NDMA removal rates were higher than those observed in single-component systems, likely due to the increased number of water pollutants and the greater complexity of interactions. The NDMA removal rate change tendency was steady and then gradually increased with an increasing volume of filtrate, reaching the highest removal rate of 42.51% at 7 L (breakthrough volume), which was 28.85–28.73% higher than that in single-component systems (DTA-NDMA, HAs-NDMA, DMA-NDMA, NH_4_NO_3_-NDMA). The corresponding highest removal rates for turbidity, COD_Mn_, DMA, and NH_3_-N were 86.24%, 38.85%, 31.03%, and 33.30%, respectively.

Filtration after enhanced coagulation (RSF-SSEC) and conventional (RSF-SSCC) coagulation was also performed for mixed multi-component systems, and the results are presented in Figure 4 and Figure 5. The removal change tendency in RSF-SSEC mirrored that in single-component systems; however, removal rates in mixed systems were higher due to the greater complexity and diversity of interactions, leading to the increased adsorption of both NDMA and water pollutants on sand surfaces. For instance, NDMA removal rates in RSF-SSEC (using PAC/PDMDAAC) and RSF-SSCC (using PAC only) increased with filtrate volume, reaching a maximum of 53.68% and 50.72%, respectively, at 6 L. The NDMA removal rates in RSF-SSEC were 11.17% and 2.96% higher than those in RSF-SSD and RSF-SSCC for mixed systems, respectively. Additionally, compared to single-component systems, the NDMA removal rate in RSF-SSEC was 36.26–38.56% higher, depending on the specific single-component system. These improvements are attributed to the higher effectiveness of the PAC/PDMDAAC composite coagulant, which enhances removal via charge neutralization and adsorption bridging effects. The coagulant’s impact is more pronounced in mixed multi-component systems due to the greater complexity of interactions. The corresponding water pollutant parameter removal rates in RSF-SSEC and RSF-SSCC for mixed multi-component systems were 87.50% and 86.70% (turbidity); 43.66% and 41.94% (COD_Mn_); 41.67% and 37.50% (NH_3_-N); and 41.90% and 53.30% (DMA), respectively, at the same breakthrough volume of 6 L.

Although RSF-SSEC provided the best overall NDMA and water pollutant removal efficiencies, they remained lower than those observed in Yangtze River raw water treatment. This difference is likely due to variations in composition, constituent diversity, and water chemistry. These results suggest that NDMA and water pollutants exhibit a coordinated removal effect on sand surfaces, with only limited competitive removal interactions.

### 2.2. NDMA and Pollutant Parameter Removal Rate Change Tendency and Their Correlation in Various Rapid Sand Filtration Water Systems

#### 2.2.1. Correlation in Rapid Sand Filtration of Simulated Supernatant Directly for Single-Component and Mixed Multi-Component Systems

A correlation analysis of NDMA and the water pollutant removal rate change tendency was conducted using both direct observation and statistical methods. Table 1 presents the statistical correlations between NDMA and pollutant removal rates in rapid sand filtration (RSF) for both single-component and mixed multi-component systems, while additional direct observation correlations are shown in Tables S1 and S2.

The order of the NDMA removal rate with the corresponding water pollutant parameter removal rate change tendency was HAs-NDMA > NH_4_NO_3_-NDMA > DMA-NDMA > DTA-NDMA > NDMA. This pattern suggests that NDMA adsorption is significantly influenced by interactions between NDMA, water pollutants, and sand surfaces. The correlation obtained by a statistical method (Table 1) showed a strong positive correlation for NDMA–humic acid, NDMA-DMA, NDMA–ammonium nitrate, and NDMA–diatomite, which was statistically significant. The correlation order in RSF-SSD for single-component systems was primarily determined by surface charge interactions between water pollutants and sand surfaces, as well as NDMA adsorption on water pollutants, which varied based on microstructure, functional groups, and polarity. For instance, the removal rate in humic acid–NDMA is the best in RSF-SSD due to the network of microstructure, available multifunctional groups, and high polarity for the increased adsorption of NDMA; NH_4_NO_3_-NDMA ranked second, likely due to the strong highly positive charges which facilitated its attachment to the sand surface, thereby enhancing NDMA adsorption; and DMA-NDMA followed, benefiting from positive charge interactions with the sand surface and the similarity in functional groups between DMA and NDMA. Diatomite–NDMA has the smallest removal rate due to limited adsorption on sand. These correlation trends align with the preliminary findings from the treatment of Yangtze River raw water using enhanced coagulation and rapid sand filtration. However, in that case, the influence of major water pollutants on NDMA removal was not explicitly quantified due to real water complexity [28].

In RSF-SSD for mixed multi-component systems, the correlation obtained by a statistical method (using Kendall method, Table 1) revealed trends similar to those observed in single-component systems, with a comparable correlation order. However, the results indicate that as water pollutant diversity increases, interactions become more complex, leading to the stronger adsorption of both NDMA and water pollutants on sand surfaces. This explains the higher removal efficiencies observed in mixed multi-component systems compared to single-component systems. Additionally, the correlation strength between NDMA and the corresponding water pollution parameters in RSF-SSD for mixed-component systems was generally stronger than that in single-component systems, aligning with the findings from Yangtze River surface water treatment by rapid sand filtration after coagulation [16,28]. The removal of NDMA by sand filtration is strongly correlated with the removal of residual suspended pollutants in both single-component and mixed multi-component systems. More importantly, the discovered correlation order varies depending on pollutant properties and coagulation function on sand surface interactions.

#### 2.2.2. Correlation in Rapid Sand Filtration of Simulated Supernatant After Coagulation for Single-Component and Mixed Multi-Component Simulation Systems

The correlation analysis between NDMA and the water pollutant removal change tendency was conducted using both direct observation and statistical methods. Table 1 presents the statistical correlations between NDMA and pollutant removal rates in the rapid sand filtration (RSF) of simulated supernatants for both single-component and mixed multi-component systems, while Tables S1 and S2 provide observations from direct analysis. The correlation order of NDMA and the water pollutant removal rate change tendency was HAs-NDMA > DTA-NDMA > DMA-NDMA > NH_4_NO_3_-NDMA > NDMA. The correlation obtained by a statistical method using Kendall tau (Table 1) showed that there was a strong positive correlation for NDMA–turbidity, NDMA-COD_Mn_, NDMA-DMA, and NDMA-NH_3_-N by the rapid sand filtration of the simulated supernatant after enhanced coagulation for single-component systems. This highlights the influence of coagulation on NDMA adsorption on both pollutants and sand surfaces, which differs slightly from direct rapid sand filtration.

For mixed multi-component systems, the correlation order in RSF-SSEC remained similar to that of the single-component system based on the removal rate and change tendency. However, the correlations were more obvious and stronger in the rapid sand filtration of the simulated supernatant after conventional and enhanced coagulation, indicating the increased interactions and adsorption of NDMA on the sand surface. Additionally, aside from residual pollutant interactions with NDMA, residual composite coagulants—due to their long polymer chains and available adsorption sites—enhanced the adsorption of both NDMA and water pollutants onto sand pores and surfaces. This accounts for the higher NDMA removal rates observed in rapid sand filtration after enhanced coagulation for mixed multi-component systems compared to direct filtration.

Therefore, the discovered correlation orders in the rapid sand filtration of the supernatant after coagulation, especially enhanced coagulation, for single-component and mixed multi-component systems are similar, with a strong correlation between NDMA and the water pollutant removal rate change tendency. However, the correlation strength was greater for mixed multi-component systems due to the complexity of interactions.

#### 2.2.3. Coordination Effects of NDMA Removal in Mixed Simulation Water Systems

In the rapid sand filtration of the simulated supernatant directly (RSF-SSD) and rapid sand filtration after enhanced coagulation (RSF-SSEC) for mixed multi-component water systems, the highest combined NDMA removal rates were 42.50% and 53.30%, respectively. However, an important observation was that the sum of the NDMA removal rate for single-component systems in both RSF-SSD and RSF-SSEC was higher than that for mixed component systems. This suggests that NDMA removal in mixed systems is not synergistic but instead follows a coordinated removal effect with limited competition when using rapid sand filtration.

It can be inferred that mixed water pollutants collectively and cooperatively influenced NDMA adsorption on the sand surface. However, the reduction in NDMA removal rates in mixed multi-component systems (compared to the sum of individual single-component systems) is likely due to the competitive adsorption of other pollutants on sand pores and surfaces [20,31]. This was in agreement with observations in previous NDMA and water quality parameters for the treatment of Yangtze River raw water by rapid sand filtration after coagulation [27]. In summary, the coordination effect in mixed multi-component water systems contributed to higher NDMA and pollutant removal rates, driven by simultaneous adsorption and surface charge interactions on the sand surface.

### 2.3. Surface Charge Changes in Single-Component and Mixed Multi-Component Systems: Insights from Zeta Potentials

**Figure 6:**
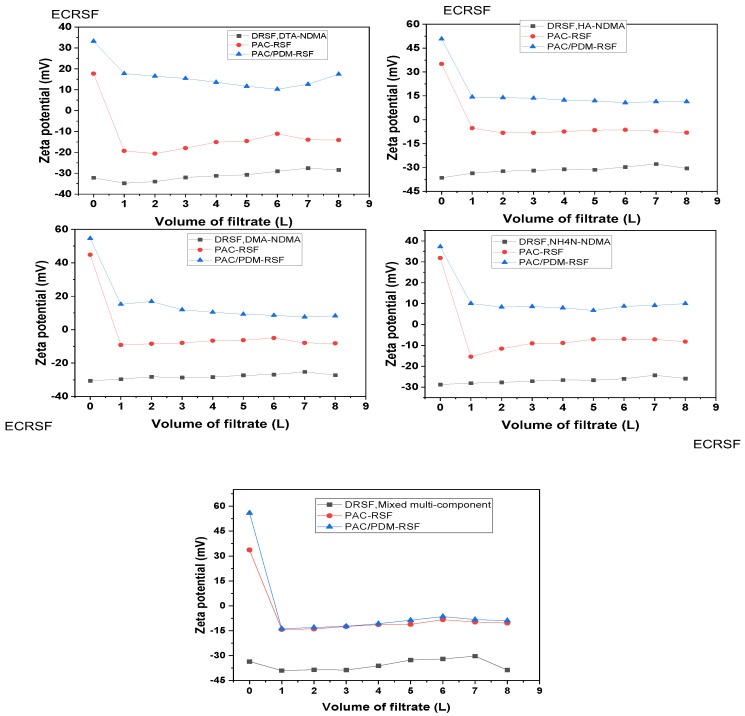
Zeta potentials at varying volumes of rapid sand filtration of simulated supernatant directly and after enhanced coagulation for various single- and mixed multi-component systems.

Figure 6 presents the zeta potential values at varying volumes in the rapid sand filtration (RSF) of simulated supernatants, both directly and after enhanced coagulation, for single-component and mixed multi-component systems. A summary of these zeta potentials is provided in Table S3.

Previous studies on water quality parameter removal using enhanced coagulation with rapid sand filtration [16,28] reported that zeta potentials after RSF reflect the attachment or detachment of residual suspended particles due to physical and chemical interactions with sand pores and surfaces. Additionally, in prior preliminary NDMA removal mechanism assumptions, it was assumed that the increased NDMA removal rate caused by rapid sand filtration may be due to surface charges alterations in residual suspended particles after the enhanced coagulation of Yangtze River raw water using composite PAC/PDMDAAC. These changes promoted particle attachment to sand pores and surfaces [27]. However, due to diverse and complex residual particle compositions, direct surface charge changes were not previously observed. Nonetheless, less negative zeta potential values were generally reported after RSF operations. In this study, zeta potentials were monitored for the RSF of simulated supernatants, both directly and after enhanced coagulation, in single-component and mixed multi-component systems containing residual pollutants.

For single-component filtrate systems, zeta potential values gradually increased (became less negative) with increasing filtrate volume (Figure 6). All zeta potential values remained negative, indicating no significant changes in water pollutant surface charges due to the RSF of the supernatant directly (RSF-SSD). This is consistent with the zeta potential trends observed in Yangtze River raw water treatment using RSF [16,28]. Furthermore, in RSF-SSD for mixed multi-component systems, zeta potential values were generally more negative than those for single-component systems, likely due to the diverse and complex composition of residual particles (water chemistry). This suggests that electrostatic repulsion dominated, as pollutants like diatomite, humic acid salt, and sand grains are negatively charged based on their zeta potential values. However, despite electrostatic repulsion, NDMA removal increased in RSF-SSD for both single-component and mixed-component systems compared to the blank system (NDMA only). This suggests that attractive interactions between water pollutants and sand surfaces played a role. The unequal charge distribution between pollutants and sand grains likely reduced electrostatic repulsion, facilitating NDMA adsorption [32,33]. Hence, the unequal charges and size of diatomite and humic acid salt with high polarity may also account for the observed adsorption on the negatively charged sand surface.

For the simulated Yangtze River raw water containing both NDMA and pollutants, zeta potentials were initially negative before coagulation. However, after coagulation with PAC and composite PAC/PDMDAAC, they shifted from negative to positive values (Figure 6). This indicated that the aluminum and ammonium positively charged ions from the coagulants neutralized the initial negative charges in simulated raw water. In RSF-SSEC, zeta potentials for DTA-NDMA, HAs-NDMA, DMA-NDMA, and NH_4_NO_3_-NDMA were 10.25 mV, 10.73 mV, 8.54 mV, and 8.72 mV, respectively, while in RSF-SSCC, the values were −11.07 mV, −6.35 mV, −5.02 mV, and −6.93 mV. While the pollutant size distribution relative to sand pore size is also important in media filtration-based removal, these results confirm that surface charge modifications—particularly after enhanced coagulation—facilitate NDMA and pollutant removal through electrostatic interactions, in addition to mechanical straining.

### 2.4. NDMA Removal Mechanism Analysis via Adsorption in Filtration Water Systems

#### 2.4.1. Adsorption Mechanism of NDMA on Water Pollutants Based on Surface Microstructure and Functional Group Analysis

The removal of NDMA by rapid sand filtration can be explained through the microstructure, functional groups, and polarity of water pollutants, which influence NDMA interactions and adsorption onto sand pores and surfaces, including surface charge interactions [34,35]. The filtration mechanism primarily involves the transport and attachment of residual suspended particles within sand pores, predominantly through electrostatic and van der Waals interactions via available functional groups [28,31,36,37]. Larger particles are typically removed by straining, while medium and smaller particles are removed via adsorption. 

In the rapid sand filtration of the simulated supernatant directly (RSF-SSD) for both single-component and mixed multi-component systems, NDMA removal rates were higher in the presence of diatomite (DTA), humic acid salt (HAs), dimethylamine (DMA), and ammonium nitrate (NH_4_NO_3_) than in the blank system (NDMA only). This suggests that NDMA adsorption occurs to varying degrees, depending on the microstructure, functional groups, and polarity of these water pollutants, which subsequently enhances NDMA attachment and removal on sand surfaces. In addition, the extent and order of NDMA adsorption onto water pollutants and sand in RSF-SSD depend on the physical and chemical properties of each component, including its molecular size, polarity, and available functional groups [38]. 

For instance, reports in the literature indicate that humic acid has a large apparent surface areas, a porous microstructure, and large molecular size relative to sand pores [39]. The porous structure, coupled with multiple adsorption sites, facilitates strong NDMA adsorption before attachment to sand surfaces. The high NDMA removal efficiency observed with HAs is attributed to its multifunctional and highly polar groups, including carboxyl (-COOH, 1.6 D), amine (-NH_2_, 1.3 D), and phenol (-OH, 1.85 D) [30]. These functional groups enhance NDMA adsorption through hydrogen bonding and dipole interactions, making humic acid salt the most effective NDMA adsorbent among the tested water pollutants. Also, diatomite (DTA) is mainly silica (90%) with trace metals and has a porous nature with functional groups like silanol (Si-OH), siloxane (Si-O-Si), and siloxide (SiO^−^) and a molecular size range of 50–200 µm [40,41] With a molecular size range of 50–200 µm, diatomite is relatively smaller than sand pores, limiting its removal by straining. However, the microstructure of diatomite and the available surface silanol groups with polar environments can interact and adsorb NDMA through hydrogen bonding and van der Waal forces. Additionally, the structural similarity between diatomite and sand enhances NDMA adsorption onto the sand surface, making it a significant, though secondary, adsorption site for NDMA.

On the other hand, DMA exhibits moderate interaction with NDMA due to its limited functional groups and adsorption sites. However, its structural similarity in terms of functional groups with NDMA may result in weak molecular interactions, such as hydrogen bonding and van der Waals forces, which may still facilitate NDMA adsorption. Ammonium nitrate (NH_4_NO_3_) is a soluble ionic compound with high polarity and small molecular size (0.3 nm). NH_4_NO_3_ can interact with NDMA through ion–dipole interactions due to the positively charged ammonium ions and dipole between the electronegative nitrogen and oxygen atoms on NDMA, but the interaction and adsorption capacity for NDMA are limited due to the ionic nature of NH_4_NO_3_, which is different from the organic nature of NDMA. However, NH_4_NO_3_, due to its strong positive ions, can be strongly adsorbed on the sand surface due to strong electrostatic interactions.

The observed adsorption order of NDMA on water pollutants and sand in RSF-SSD is as follows: humic salt–NDMA > ammonium nitrate–NDMA > dimethyl amine–NDMA > diatomite–NDMA. In conclusion, the NDMA adsorption order varies significantly across different water pollutants and sand, depending on their microstructure, functional groups, and polarity. The presence of specific pollutants enhances NDMA removal in rapid sand filtration, primarily through adsorption, charge interactions, and structural compatibility with sand pores and surfaces.

#### 2.4.2. Adsorption of NDMA Directly on Sand with Both NDMA and Water Pollution Parameters Based on Microstructure, Functional Group, and Surface Charge Interactions

In the rapid sand filtration of the simulated supernatant directly (RSF-SSD) for a blank system containing NDMA in aqueous solution, the NDMA removal rate was low. This is attributed to NDMA’s strong affinity for water, neutral nature, and small molecular size (0.45 nm) compared to sand grains (0.6–0.8 mm) [29,42,43] The weak interaction between NDMA and sand surfaces further limits adsorption and removal, as reported in previous studies [22,31,44]. 

However, in RSF-SSD with single-component and mixed multi-component systems, NDMA adsorption varied based on the microstructure, functional groups, and polarity of coexisting water pollutants. Humic acid salt (HAs) exhibited the highest level of NDMA adsorption, followed by diatomite (DTA), due to its porous nature and surface functional groups. Dimethylamine (DMA) showed moderate adsorption, while ammonium nitrate (NH_4_NO_3_) had the lowest due to its incompatibility with NDMA. In contrast, the observed order of the NDMA removal rate change tendency was HAs-NDMA > NH_4_NO_3_-NDMA > DMA-NDMA > DTA-NDMA > NDMA (blank system). This trend suggests that beyond direct NDMA adsorption on water pollutants, the subsequent attachment of both NDMA and pollutants to sand surfaces via surface charge interactions plays a crucial role. Thus, NDMA removal in RSF-SSD is governed not only by microstructure, functional groups, and polarity but also by surface charge interactions among diatomite, humic acid salt, dimethylamine, ammonium nitrate, and sand [35,38]. 

**Figure 7:**
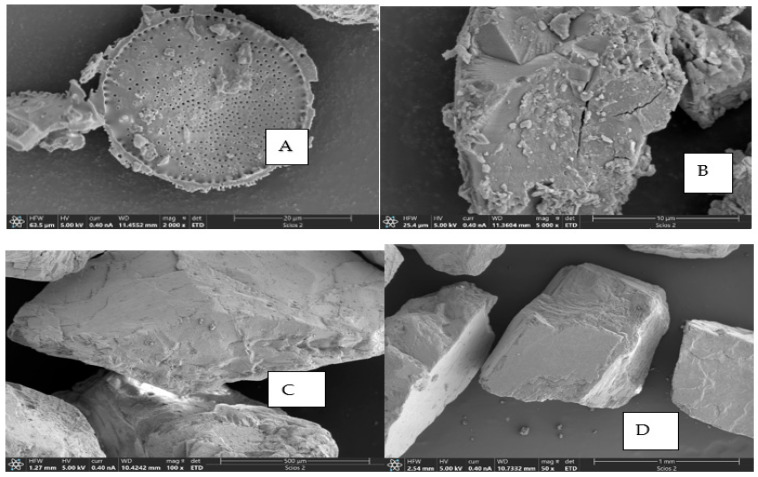
SEM micrographs (**A**–**D**) representing diatomite, mixed multi-component system, rapid sand filtration after enhanced coagulation of HAs-NDMA, and sand before use.

For instance, diatomite (DTA) is mainly silica (90%) with some trace metals, and it has a porous nature with functional groups like silanol (Si-OH), siloxane (Si-O-Si), and siloxide (SiO^−^), as well as net charges in water that are negative, the same as sand grain in water, and a molecular size range of 50–200 µm [40,41]. Although electrostatic repulsion is expected between diatomite and sand, van der Waals forces can override repulsion at short distances, facilitating NDMA adsorption [21,45] This is supported by zeta potential values in DTA-NDMA RSF-SSD (−32.16 mV for diatomite and −13 mV for sand), which indicate charge polarization, with sand acting as a relatively positive site for adsorption [32,33]. Furthermore, NDMA’s nitrosyl group (N=O) enables ion–dipole and hydrogen bonding interactions with surface silanol groups on both diatomite and sand [46]. SEM micrographs (Figure 7) confirm the strong adsorption of nanosized diatomite pores onto sand, further enhancing NDMA adsorption.

In contrast, dimethyl amine (DMA) and ammonium nitrate (NH_4_NO_3_) with amino and nitro groups, respectively, have a net positive charge in aqueous water, supported by zeta potentials with a less negative value in DMA-NDMA (−30.64 mV) and NH_4_NO_3_-NDMA (−28.75 mV) in RSF-SSD single-component systems. The molecular sizes of DMA and NH_4_NO_3_, respectively, are 0.56 µm and 0.30 nm, which are relatively smaller than sand pore size, and this implies that their removal via the straining mechanism is limited. However, the direct removal of DMA and NH_4_NO_3_ is expected to be due to strongly electrostatic attractions, but the NDMA removal rate was slightly better in RSF-SSD for DMA-NDMA than NH_4_NO_3_-NDMA. This may be due to DMA having functional groups similar to those of NDMA, resulting in stronger interactions and a stronger adsorption of both DMA and NDMA on the sand surface, while NH_4_NO_3_ may have weaker interactions with NDMA due to an incompatible nature but stronger adsorption on the sand surface due to electrostatic interactions. Both possess amino and nitro groups, leading to a net positive charge in aqueous solutions, as reflected in their zeta potentials (−30.64 mV for DMA-NDMA and −28.75 mV for NH_4_NO_3_-NDMA). Their molecular sizes (DMA: 0.56 µm; NH_4_NO_3_: 0.30 nm) are too small for removal by straining. However, DMA exhibits stronger NDMA adsorption than NH_4_NO_3_ due to its structural similarity, which enhances intermolecular interactions. NH_4_NO_3_, despite its weaker NDMA affinity, is strongly adsorbed onto sand via electrostatic interactions.

Therefore, NDMA-HAs with bigger molecular size relative to sand pore size and multifunctional groups is removed first by straining and adsorption, and then NDMA-DMA and NDMA-NH_4_NO_3_ with positive charges are removed, followed by NDMA-DTA with negative charges for adsorption to sand pores and the surface, while NDMA only has the lowest levels of compatibility and adsorption on the sand surface due to its high solubility and smaller size [4,20,28]. The adsorption functions of both NDMA and water pollutants, including direct NDMA adsorption on the sand surface, are depicted in Figure S5.

The NDMA removal mechanism in mixed multi-component systems mirrors that of single-component systems but exhibits more diverse and complex interactions, enhancing overall adsorption onto sand pores and surfaces. This observation aligns with previous NDMA adsorption assumptions [27], but adsorption variability was not observed in real raw water. Ultimately, NDMA and pollutant adsorption on sand depends on microstructure, functional groups, polarity, and surface charge interactions.

The experimental results indicate that NDMA removal in the rapid sand filtration of the simulated supernatant after enhanced coagulation (RSF-SSEC) varies based on system composition—blank, single-component, or mixed multi-component—providing mechanistic insights. The use of a composite PAC/PDMDAAC coagulant, compared to PAC only, influences NDMA removal by altering pollutant interactions and adsorption dynamics.

In the rapid sand filtration of the simulated supernatant after enhanced coagulation (RSF-SSEC) for a blank system (NDMA only in aqueous solution), NDMA removal (12.84%) was higher than that in RSF-SSD (10.29%). This improvement is attributed to residual coagulant with positive charges, which enhances electrostatic and van der Waals interactions with sand surfaces, facilitating NDMA attachment and removal.

In the rapid sand filtration of the simulated supernatant after enhanced coagulation (RSF-SSEC) for single-component systems, the NDMA removal mechanism differed from that in RSF-SSD due to the effects of the composite PAC/PDMDAAC coagulant. The composite coagulant demonstrated superior removal efficiency over PAC alone, consistent with the literature findings attributing this to high cationic charge density and long polymer chains providing multiple adsorption sites [15,28]. Firstly, the highly positive PAC/PDMDAAC coagulant alters pollutant surface charges, as shown by zeta potential shifts from negative to positive values (Figure 6). This facilitates the migration and attachment of pollutants such as diatomite, humic acid salt, dimethylamine, and ammonium nitrate onto sand surfaces, enhancing NDMA adsorption. Secondly, some NDMA may absorb on previously retained residual water pollutants and coagulant onto sand pores and surfaces, further promoting NDMA attachment through electrostatic attraction and van der Waals forces. Unlike previous studies on Yangtze River raw water treatment, where the zeta potential remained negative throughout the process, this study reveals a significant role of the residual coagulant in modifying adsorption behavior [28,47].

The NDMA removal mechanism in the rapid sand filtration of the simulated supernatant after enhanced coagulation (RSF-SSEC) in mixed multi-component systems followed a pattern similar to that in single-component systems but was more complex due to diverse interactions among pollutants, coagulant, and sand. The composite PAC/PDMDAAC coagulant effect was more pronounced, as evidenced by zeta potential values (Figure 6), indicating enhanced pollutant compatibility with sand surfaces. The positively charged residual coagulant first adsorbs onto pollutants during coagulation, then onto sand grains, improving pollutant and NDMA attachment [48].

Furthermore, SEM images (Figure 7) showed traces of residual composite coagulant PAC/PDMDAAC on sand grains, supporting the assumptions that the residual coagulant enhances NDMA adsorption. Improved compatibility between sand surfaces and pollutants—via pore size, surface charge, and functional groups—further increases NDMA removal through electrostatic and van der Waals interactions [25,44].

Previous studies on Yangtze River raw water treatment assumed that composite PAC/PDMDAAC initially removed some pollutants and NDMA through charge neutralization and polymer bridging flocculation, with additional NDMA adsorption occurring during filtration [27,28]. This study confirms these assumptions, demonstrating that in RSF-SSEC, NDMA removal depends not only on microstructure, functional groups, and surface charge interactions but also on coagulant function—particularly enhanced coagulation—which further improves NDMA removal efficiency.

## 3. Materials and Experimental Methods

### 3.1. Materials

#### 3.1.1. Selection of Materials

According to the design arrangements in the Introduction and based on the new insights gained from our group’s study on the NDMA removal mechanism enabled by enhanced coagulation, specific reagents and materials were selected as representative water pollutants. A stock NDMA standard (20 mg/mL at 99% purity) was used to prepare NDMA-spiked solutions, while diatomite (DTA), humic acid salt (HAs), dimethyl amine (DMA), and ammonium nitrate (NH_4_NO_3_) were selected as representative turbidity, and organic matter, NDMA precursor, and inorganic nitrogen parameters, respectively. Unlike actual reference supernatants from Yangtze River raw water, which contain a complex and unidentifiable mixture of residual suspended particles, this approach allowed for a more controlled and quantifiable analysis of NDMA removal mechanisms. To achieve this, two types of simulated supernatants—directly and after enhanced coagulation—were formulated and divided into blank, single-component, and mixed multi-component filtration systems.

The residual suspended particles were represented by diatomite (DTA) and humic acid salt (HAs), while the typical NDMA precursors in real natural waters due to the discharge of industrial and domestic wastewater were represented by dimethylamine and ammonium nitrate. For coagulation pre-treatment, conventional and enhanced coagulation were conducted using 10% polyaluminum chloride (PAC) and a composite poly-dimethyldiallylammonium chloride (PAC/PDMDAAC) coagulant (intrinsic viscosity: 1.50 dL/g; mass ratio: 10:1), respectively. Rapid sand filtration was performed using sand grains (ES = 0.6–0.8 mm) and glass filter columns (50 cm length, 1.8 cm internal diameter), following established methodologies [16,28].

#### 3.1.2. Preparation of Materials

##### Preparation of Coagulants

Based on the selection of materials, the appropriate conventional coagulant, polyaluminum chloride (PAC), and enhanced composite coagulant PAC/poly dimethyl-diallylammonium chloride (PAC/PDMDAAC) with an intrinsic viscosity of 1.50 dL/g used as the measure of polymer molecular weight, with a mass ratio (PAC to PDMDAAC) of 10:1, were prepared according to Olukowi et al. [28] and Zhou et al. [27] for the simulated supernatant after the enhanced coagulation of Yangtze River raw water.

##### Determination of Initial Conditions for Supernatants in Rapid Sand Filtration

Based on an experimental design, the initial concentrations of selected representative water pollution parameters in two types of supernatants—directly simulated and post-enhanced coagulation—were determined to mimic actual supernatants from the Yangtze River in summer (Table 2). This process involved several experimental trials to determine the initial amount of DTA, HAs, NH_4_-NO_3_, DMA, and NDMA needed to formulate the simulated supernatant for various water systems. First, varying amounts of diatomite and humic acid salt, representing turbidity and organic matter pollution, were added to 100 mL of deionized water, while ammonium nitrate and DMA concentrations were calculated based on the actual reference supernatant values in Table 2 Each formulation was spiked with 80 ng/L of NDMA to ensure homogeneity.

Secondly, conventional and enhanced coagulation treatments were conducted using PAC and composite PAC/PDMDAAC (optimal dosage: 2.6 mg/L) in jar test machines with 1 L of simulated Yangtze River raw water. The resulting supernatants were analyzed for NDMA and water pollutant parameters, and those closest to the actual reference supernatant values were selected for rapid sand filtration studies. The amounts of representative water pollutants and NDMA concentrations in the two kinds of simulated supernatants for blank, various single-component, and mixed multi-component water systems are represented in Table 2.

#### 3.1.3. Formulation of Simulated Supernatants Directly and After Enhanced Coagulation in Simulated Raw Water Systems

According to the design arrangement in the Section 1, the water pollutant parameters in the actual reference supernatants, and the selected amounts of each pollutant in Table 1, two kinds of supernatants simulated directly and after coagulation separated into blank, various single-components, and mixed multi-component water systems were prepared for rapid sand filtration operations. Briefly, the process was as follows:

(1) Blank water systems: A total of 80 µL of NDMA working standard from 0.1 g/L NDMA standard solution with a concentration of 80 µg/L was used in 100 mL of a volumetric flask. Then, 10 mL of the NDMA working standard was withdrawn with a micropipette, diluted by 1000-fold, in 10 L of deionized water to obtain the simulated supernatant water sample directly with an 80 ng/L final concentration for blank systems, while the simulated Yangtze River raw water with a 100 ng/L final concentration was also prepared following the above procedure for the supernatant after coagulation.
(2) Single-component systems: Based on the summary in Table 1, the appropriate amount of each water pollution parameter material was weighed and dissolved in 13 L of deionized water spiked with either 80 ng/L or 100 ng/L of NDMA, respectively, for simulated supernatants directly and after coagulation. The various single-component water systems were homogenized and then allowed to stand for 20 min. DTA-NDMA, HAs-NDMA, DMA-NDMA, and NH_4_NO_3_-NDMA were used for rapid sand filtration directly and rapid sand filtration after enhanced coagulation operations.
(3) Mixed multi-component systems: Based on the summary in Table 1, an appropriate amount of all water pollution parameters, namely diatomite (DTA), humic acid salt (HAs), dimethyl amine (DMA), and ammonium nitrate (NH_4_NO_3_), was weighed and dissolved in 13 L of deionized water spiked with either 80 ng/L or 100 ng/L of NDMA, respectively, for the supernatants simulated directly and after coagulation. The mixed multi-component water systems were homogenized and then allowed to stand for 20 min, which were used for rapid sand filtration directly and rapid sand filtration after enhanced coagulation.

### 3.2. Water Pollution Parameters and NDMA in Simulated Supernatants in Rapid Sand Filtration Directly and After Enhanced Coagulation Operations

#### 3.2.1. Selection and Set-Up of Rapid Sand Filters

Rapid sand filtration was conducted under previously established conditions for enhanced coagulation coupled with sand filtration in Yangtze River water studies. The filter column parameters and set-up followed protocols from earlier research [15,28].

#### 3.2.2. Water Pollutants and NDMA Removal by Coagulation of Simulated Raw Water Operations

The conventional and enhanced coagulation simulated supernatants were created in jar test machines using the formulated simulated Yangtze River raw water for blank and various single- and mixed multi-component raw water systems at an optimum dosage of 2.6 mg/L. Coagulation operations were performed according to the detailed description given by Zhou et al. [15].

#### 3.2.3. Water Pollution and NDMA Removal by Rapid Sand Filtration Directly and After Enhanced Coagulation Operations

The supernatants after the coagulation of simulated Yangtze River raw water were formulated using PAC and composite PAC/PDMDAAC, respectively, in jar test machines for blank, various single-component, and mixed multi-component formulated raw water systems at the optimum dosage of 2.6 mg/L selected based on previous NDMA removal studies. The conventional and enhanced coagulation operations were conducted according to Zhou et al. [15], while the direct simulated supernatants were directly formulated based on the appropriate amount of water pollution parameter, as highlighted in Table 2. The two kinds of supernatants were passed through prepared rapid sand filters for filtration operations. The resulting filtrates were analyzed for NDMA and other water pollutant parameters (turbidity, COD_Mn_, DMA, NH_3_-N) in various water systems. In all cases, the filtration operations were repeated at least twice for reproducibility and to ensure operational validation.

### 3.3. Basic Water Pollution Parameters and NDMA Instruments and Measurements

#### 3.3.1. Basic Water Pollution Parameter Analysis

The basic instruments used are as follows: a scattering turbidimeter (Qs 201, Suzhou, Qingan Instrument Co., Ltd., Suzhou, Jiangsu, China); a Six-Beaker program-controlled jar testing machine (TA6-2, Wuhan Henling Technology Co., Ltd., Wuhan, Hubei, China); and a double-beam spectrophotometer, zeta meter (JS 94 H, Shanghai Digital Technic Apparatus Co., Ltd., Shanghai, China). The turbidity measurement method was conducted using the scatter light turbidimeter; the CODMn measurement was conducted via the acidic potassium permanganate titration method; and the ammonia nitrogen (NH3-N) and DMA measurement methods were performed by using the Nessler reagent and copper bis (di- thiocarbamate) complex spectrophotometry, respectively [11,49,50], while the zeta potentials were monitored for surface charge changes. All water pollutant parameters were measured in triplicate, and the mean values and standard deviation were recorded.

#### 3.3.2. NDMA Measurements

The NDMA analysis was based on the methods reported in the literature involving membrane filtration, solid-phase extraction (SPE), nitrogen gas concentration, and HPLC detection [25,26,51]. Briefly, an SPE instrument was used for the pre-treatment and concentration of water samples (Model 57250, Supelco Corp., Bellefonte, PA, USA), and an HPLC-UV instrument (Agilent Technologies, 1200 infinity series, Wald Bronn, Germany) was used for NDMA detection. A C-18 Zorbax (4.6 × 250 mm, 5 µm) was employed for separation and detection. The mobile phase was composed of solvent A (water) and solvent D (methanol). The solvent gradient was initially run at 50:50 for 1 h and then at 95:5 for 1 h, which was maintained throughout the analysis, and usually, re-equilibration was carried out before the next injection. The wavelength, column temperature, and injection volume were 228 nm, 30 °C, and 20 µL, respectively. The flow rate was maintained at 1.0 mL/min. The sensitivity and selectivity of the HPLC-UV chromatograph used to effectively identify NDMA were validated with a method precision of ±2.1% and a percentage recovery in the range of 70–130%, which was in agreement with the literature [51].

The correlation between NDMA and water pollution parameter removal rates and the change tendency seen via direct observation is based on removal change tendency, the highest removal rate, and breakthrough volume. This was further verified and compared to the correlation obtained using a statistical method for the strength of correlation and significance.

#### 3.3.3. Scan Electron Microscope Analysis

The Scan Electron Microscope (SEM) model Hitachi Regulus 8100 was used to assess the surface morphology of sand before and after the filtration of DTA-NDMA, HAs-NDMA, and the simulated supernatant after enhanced coagulation in a mixed multi-component system. The accelerating voltage of the SE detector was set to 20 kV. SEM micrographs of diatomite–NDMA, mixed multi-component system, humic acid salt–NDMA obtained by rapid sand filtration after enhanced coagulation, and unused sand are presented in Figure 7.

## 4. Conclusions

This study investigated the NDMA removal mechanism via adsorption onto select water pollutants and their removal in the rapid sand filtration of simulated supernatants, both directly and after enhanced coagulation, for blank, single-component, and mixed multi-component water systems. The following conclusions were obtained:

- There was a positive correlation for NDMA-HAs, NDMA-DMA, NDMA-NH_4_NO_3_, and NDMA-DTA in both the direct and after enhanced coagulation filtration systems. The correlation order was discovered to vary to different degrees depending on the microstructure, polar functional function, and surface charges of pollutants, as well as coagulation functions on sand pores and surfaces.
- The highest NDMA removal rates in the blank system obtained using RSF directly and after the enhanced coagulation of simulated raw water were 10.29% and 12.84%, indicating that the direct adsorption of NDMA onto sand and the effects of coagulant functions increased the NDMA removal rate. In single-component systems, NDMA removal varied between 13.66 and 14.90% (direct filtration) and 15.12–21.03% (after enhanced coagulation), influenced by pollutant properties and enhanced by coagulation.
- The highest NDMA removal rates in mixed multi-component systems were 42.50% (direct filtration) and 53.30% (after coagulation), surpassing those in single-component systems. This increase highlights the synergistic adsorption effects among pollutants, though competitive interactions on sand surfaces were limited.
- The adsorption analysis conducted via the surface morphology, functional groups, and polarity of water pollutant, alongside coagulation-induced surface charge modifications, revealed their collective role in enhancing NDMA adsorption.
- The NDMA removal mechanism assumption via adsorption was verified. Initially, NDMA adsorption onto major water pollutants depends on their microstructure, polar functional groups, and surface charge interactions with sand surfaces with limited competitive removal effects. However, coagulation—particularly enhanced coagulation—further strengthens the adsorption of both NDMA only and NDMA with pollutants on sand surfaces, improving overall removal efficiency. This study provides new insights into the NDMA removal mechanisms enabled by rapid sand filtration and encourages the wider application of enhanced coagulation coupled with filtration as a viable and sustainable technology for NDMA removal, benefiting researchers and water professionals.

## Figures and Tables

**Table 1:** Kendall tau correlation analysis in rapid sand filtration of simulated supernatants directly and after enhanced coagulation for single- and mixed multi-component systems.

Rapid Sand Filtration Directly and After Enhanced Coagulation for Single-Component System				
N = 9	NDMA-DTA	NDMA-HAs	NDMA-DMA	NDMA-NH_4_NO_3_
DTA, turbidity– RSF-SSD	ŧ_b = 0.889, *p* = 0.001			
RSF-SSEC	ŧ_b = 0.833, *p* = 0.002			
HAs, COD_Mn_– RSF-SSD		ŧ_b = 0.9845, *p* = 0.000		
RSF-SSEC		ŧ_b = 0.889, *p* = 0.001		
DMA– RSF-SSD			ŧ_b = 0.915, *p* = 0.000	
RSF-SSEC			ŧ_b = 0.889, *p* = 0.001	
NH_4_NO_3_– RSF-SSD				ŧ_b = 0.944, *p* = 0.001
RSF-SSEC				ŧ_b = 0.915, *p* = 0.001
**Rapid Sand Filtration Directly and After Enhanced Coagulation for Mixed Multi-Component System**				
DTA, turbidity– RSF-SSD	ŧ_b = 0.786, *p* = 0.000			
RSF-SSEC	ŧ_b = 1.000 *p* = 0.000			
HAs, COD_Mn_ RSF-SSD		ŧ_b = 0.994, *p* = 0.000		
RSF-SSEC		ŧ_b = 1.000, *p* = 0.000		
DMA RSF-SSD			ŧ_b = 0.800, *p* = 0.003	
RSF-SSEC			ŧ_b = 1.000, *p* = 0.000	
NH_4_NO_3_ RSF-SSD				ŧ_b = 0.868, *p* = 0.001
RSF-SSEC				ŧ_b = 0.915, *p* = 0.036

Note: DTA, HAs, DMA, and NH_4_NO_3_ represent diatomite (turbidity), humic acid salt (COD_Mn_), dimethyl amine (DMA), and ammonium nitrate salt (NH_3_-N, respectively. RSF-SSD and RSF-SSEC represent rapid sand filtration of simulated supernatant directly and rapid sand filtration of simulated supernatant after enhanced coagulation. Significance level was (*p* = 0.01), and correlation was significant in all cases.

**Table 2:** Water pollution parameter materials and NDMA concentrations in simulated supernatant directly and after enhanced coagulation of simulated raw water as filtration feeding water sample.

	DTA (g/L) Turbidity	HAs (g/L) COD_Mn_	NH_4_NO_3_/(g/L)	DMA (g/L)	NDMA/(ng/L)
Direct simulated supernatant	0.01	0.0022	0.10	0.001	80.00
Simulated supernatant after enhanced coagulation	0.06	0.0024	0.16	0.001	100.00

Note: DTA, HAs, DMA, and NH_4_NO_3_ represent diatomite (turbidity), humic acid salt (COD_Mn_), dimethyl amine (DMA), and ammonium nitrate salt (NH_3_NO_4_), respectively.

## Data Availability

The authors declare that data will be made available on request.

## Supplementary Materials

The following supporting information can be downloaded at https://www.mdpi.com/article/10.3390/molecules30102094/s1, Table S1: Correlation obtained by observation between water pollution parameters and NDMA removal regulations in rapid sand filtration of simulated supernatants for single-component systems, Table S2: Correlation between water pollution parameters and NDMA removal regulation in rapid sand filtration of simulated supernatants for mixed multi-component systems, Table S3: Zeta potential values in rapid sand filtration in simulated supernatant directly and after enhanced coagulation for single- and mixed multi-component systems, Figure S1: Water pollution parameter regulations in rapid sand filtration of simulated supernatant directly for various single-component systems, Figure S2: NDMA removal regulations in rapid sand filtration of simulated supernatant directly for various single-component systems, Figure S3: Water pollution parameter removal regulations in terms of rapid sand filtration of simulated supernatant directly for single-component and mixed multi-component systems, Figure S4: NDMA removal regulations in rapid sand filtration of simulated supernatant directly for single-component and mixed multi-component systems, Figure S5: NDMA and water pollution parameter interactions and NDMA removal mechanism enabled by rapid sand filtration of simulated supernatant directly for blank and single-component systems.
