# Supplementary material for: Mechanistic Insights into NDMA Adsorption onto Selected Pollutants and Their Removal via Direct Rapid Sand Filtration and After Enhanced Coagulation
Source: Molecules. 2025 May 8;30(10):2094. doi: 10.3390/molecules30102094 (PMC12113702; doi:10.3390/molecules30102094)
Supplement: Supplementary file 1 [file molecules-30-02094-s001.zip › molecules-3582421-supplementary.pdf]

Supplemental data

Supplemental Tables

Table S1: Correlation by observation between water pollution parameters and NDMA removal regulations in rapid sand filtration of simulated supernatants for single component systems

	Direct rapid sand filtration			Rapid sand filtration after conventional coagulation			Rapid sand filtration after enhanced coagulation		
	Residual value at max. removal	Max. removal rate (%)	Change tendency	Residual value at max. removal	Max. removal rate (%)	Change tendency	Residual value at max. removal	Max. removal rate (%)	Change tendency
DTA Turbidity (NTU)	0.20	82.14 %	Sharp increase, then gradual increase	0.21	80.90 %	Sharp increase, then gradual increase	0.18	82.86	Sharp increase, then gradual increase
DTA-NDMA (ng/L)	72.22	13.66 %	Gradual increase with increasing vol. of filtrate	78.58	15.54 %	Gradual increase with increasing vol. of filtrate	78.30	17.42 %	Gradual increase with increasing vol. of filtrate
HAs (COD_{Mn}) mg/L	0.90	26.82 %	Gradual increase reaching max. at 7 L	0.80	33.30 %	Gradual increase reaching max. at 6L	0.70	39.23 %	Gradual increase reaching max. at 6L
HAs-NDMA (ng/L)	72.93	14.90 %	Gradual increase	77.72	18.60 %	Gradual increase	73.56	21.03 %	Gradual increase
DMA (mg/L)	0.21	25.00 %	Gradual increase	0.23	23.30 %	Gradual increase	0.22	31.25 %	Gradual increase
DMA-NDMA (ng/L)	73.65	14.59	Gradual increase	84.65	14.28 %	Gradual increase	83.28	15.45 %	Gradual increase
NH_3N (mg/L)	0.03	62.50	Gradual increase	0.03	70.00 %	Gradual increase	0.12	40.00 %	Gradual increase
NH_4NO_3-NDMA (ng/L)	70.85	13.78	Gradual increase	83.23	13.37	Gradual increase	78.99	15.12 %	Gradual increase
NDMA only (ng/L)	72.86	10.29	Gradual increase	84.70	11.19 %	Gradual increase	81.38	12.84 %	Gradual increase

Note: DTA, HAs, DMA, and NH_4NO_3, respectively represents diatomite (turbidity), humic acid salt (organic matter), dimethyl amine (NDMA precursor), ammonium nitrate (NH_3-N).

Table S2: Correlation between water pollution parameters and NDMA removal regulation in rapid sand filtration of simulated supernatants for mixed multi-component systems

	Direct rapid sand filtration			Rapid sand filtration after conventional coagulation			Rapid sand filtration after enhanced coagulation		
	Residual value at max. removal	Max. removal rate (%)	Change tendency	Residual value at max. removal	Max. removal rate (%)	Change tendency	Residual value at max. removal	Max. removal rate (%)	Change tendency
DTA Turbidity (NTU)	0.15	86.24 %	Sharp increase, then gradual increase	0.14	86.70 %	Sharp increase, then gradual increase	0.13	87.50 %	Sharp increase, then gradual increase
DTA-NDMA (ng/L)	69.56	42.51 %	Gradual increase with increasing vol. of filtrate	53.23	50.72 %	Gradual increase with increasing vol. of filtrate	50.01	53.68 %	Gradual increase with increasing vol. of filtrate
HAs (COD_{Mn}) mg/L	0.90	38.35 %	Gradual increase reaching max. at 7L	0.90	41.94 %	Gradual increase reaching max. at 6L	0.80	43.66 %	Gradual increase reaching max. at 6L
HAs-NDMA (ng/L)	69.56	42.51 %	Gradual increase	53.23	50.72 %	Gradual increase	50.01	53.68 %	Gradual increase
DMA (mg/L)	0.20	31.03 %	Gradual increase	0.15	37.50 %	Gradual increase	0.14	41.67 %	Gradual increase
DMA-NDMA (ng/L)	69.56	42.51 %	Gradual increase	53.23	50.72 %	Gradual increase	50.01	53.68 %	Gradual increase
NH_3-N (mg/L)	0.08	33.30 %	Gradual increase	0.07	53.30 %	Gradual increase	0.18	41.94 %	Gradual increase
NH_4NO_3-NDMA (ng/L)	69.56	42.51 %	Gradual increase	53.23	50.72 %	Gradual increase	50.01	53.68 %	Gradual increase
NDMA only (ng/L)	80.93	10.29	Gradual increase	102.46	11.19 %	Gradual increase	99.34	12.84 %	Gradual increase

Note: DTA, HAs, DMA, and NH_4N, respectively represents diatomite (turbidity), humic acid salt (organic matter), dimethyl amine (NDMA precursor), ammonium nitrate (NH_3-N).

Table S3: Zeta potential values in rapid sand filtration in simulated supernatant directly and after enhanced coagulation for single and mixed multi-component systems

Rapid sand filtration of simulated supernatant directly for single and mixed multi-component systems zeta potential (mV)					
	Diatomite (DTA-NDMA)	Humic acid salt (HAs-NDMA)	Dimethylamine (DMA-NDMA)	Ammonium nitrate (NH4N-NDMA	Mixed multi-component
DSSW	-31.16	-36.45	-30.64	-28.75	-33.56
RSF-SSD at 7 L	-27.58	-27.84	-25.23	-23.28	-30.25
Rapid sand filtration after conventional coagulation for single and mixed multi-component zeta potential (mV)					
SRW	-35.02	-43.86	-35.41	-24.14	-34.22
SSCCW	17.70	35.06	44.82	31.79	33.63
RSF-SSCC at 6 L	-11.07	-6.35	-5.02	-5.93	-8.32
Rapid sand filtration after enhanced coagulation for single and mixed multi-component systems zeta potential (mV)					
SRW	-34.17	-45.12	-35.87	-27.06	-35.94
SSECW	33.22	50.77	54.64	37.25	55.89
RSF-SSEC at 6 L	10.25	10.73	8.54	8.72	-7.43

Note: SRW, SSDW, SSCCW, SSECW, respectively, represents simulated raw water, simulated supernatant directly water, simulated supernatant water after conventional coagulation, and enhanced coagulation while RSF represent- rapid sand filtration.

Supplemental Figures

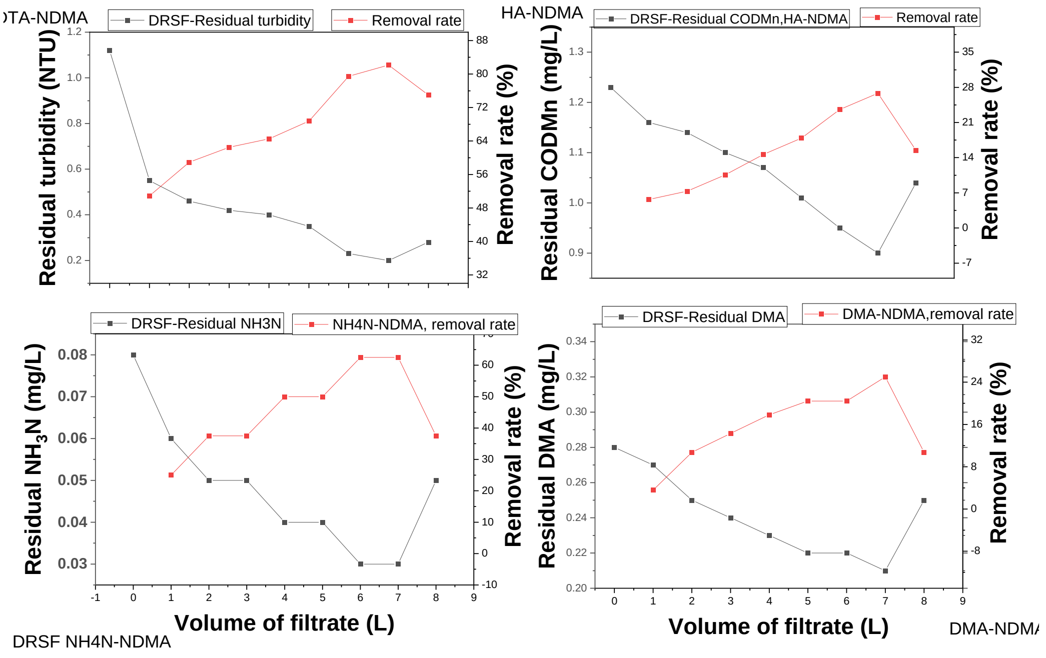

Figure S1: Water pollution parameter regulations in rapid sand filtration of simulated supernatant directly for various single component system

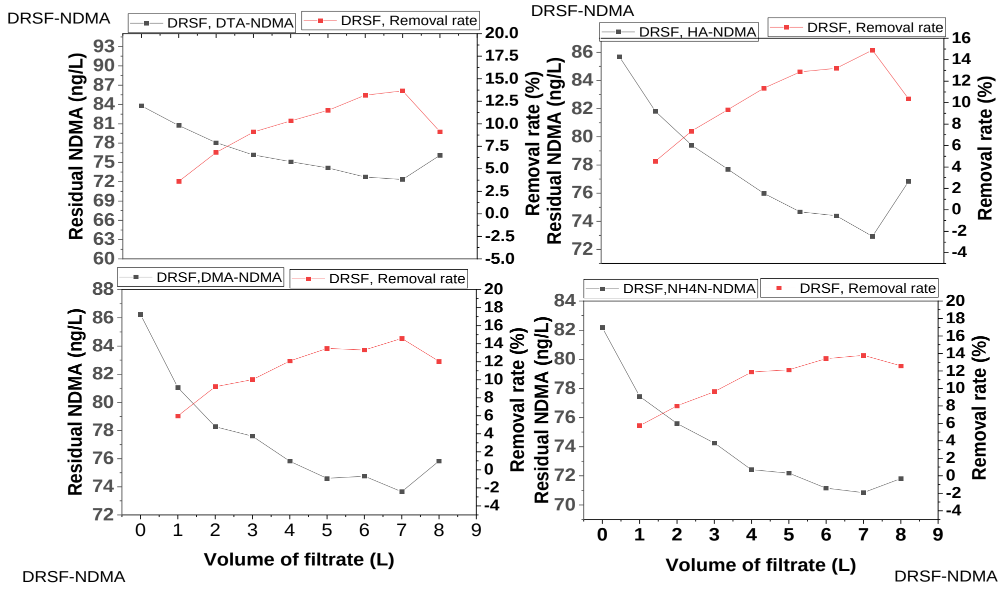

Figure S2: NDMA removal regulations in rapid sand filtration of simulated supernatant directly for various single component system

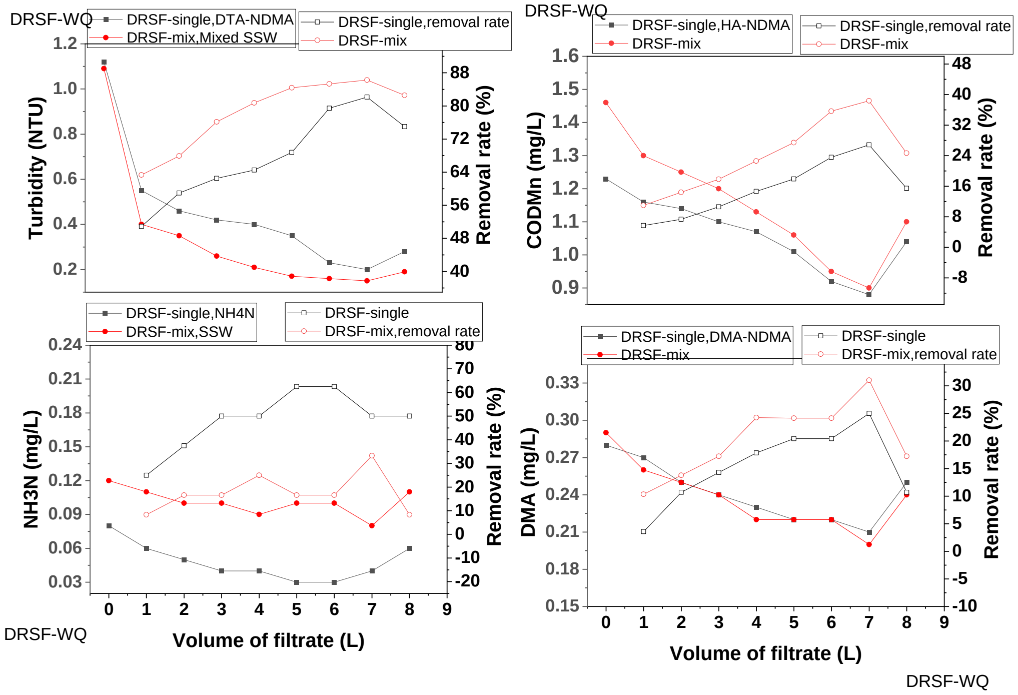

Figure S3: Water pollution parameters removal regulations in rapid sand filtration of simulated supernatant directly for single component and mixed multi-component systems

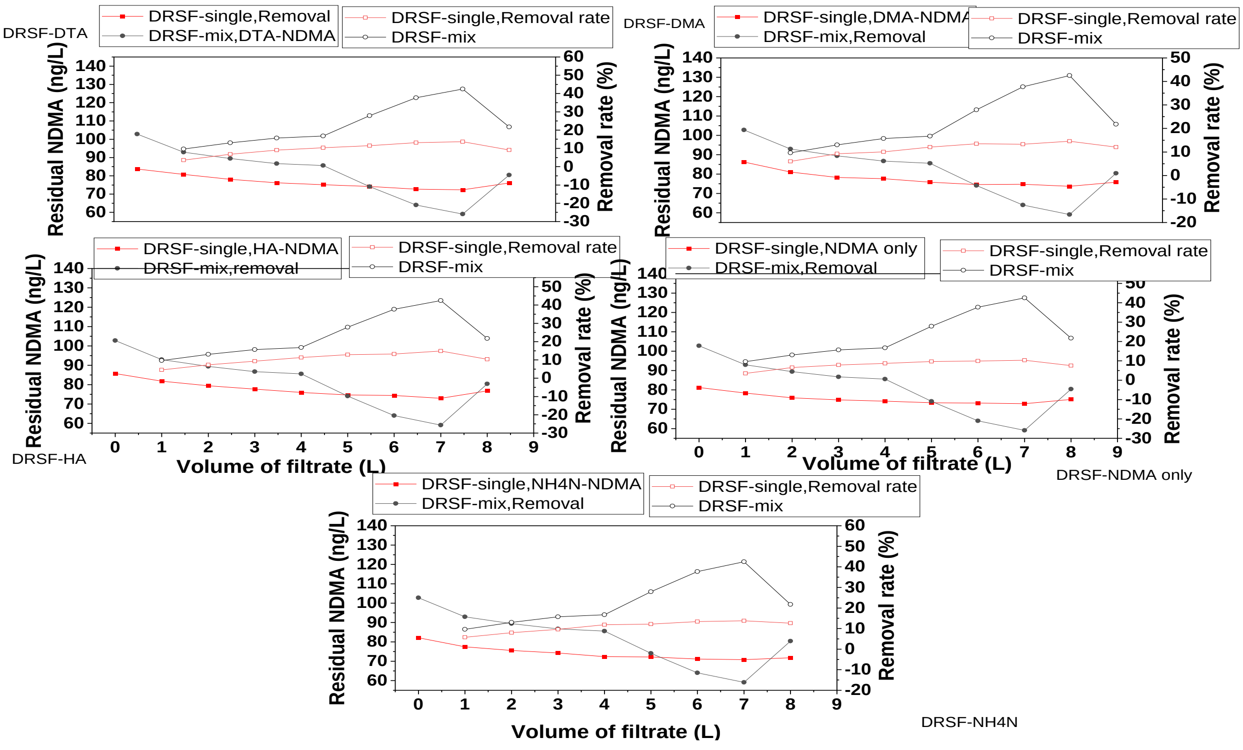

Figure S4: NDMA removal regulations in rapid sand filtration of simulated supernatant directly for single component and mixed multi-component system

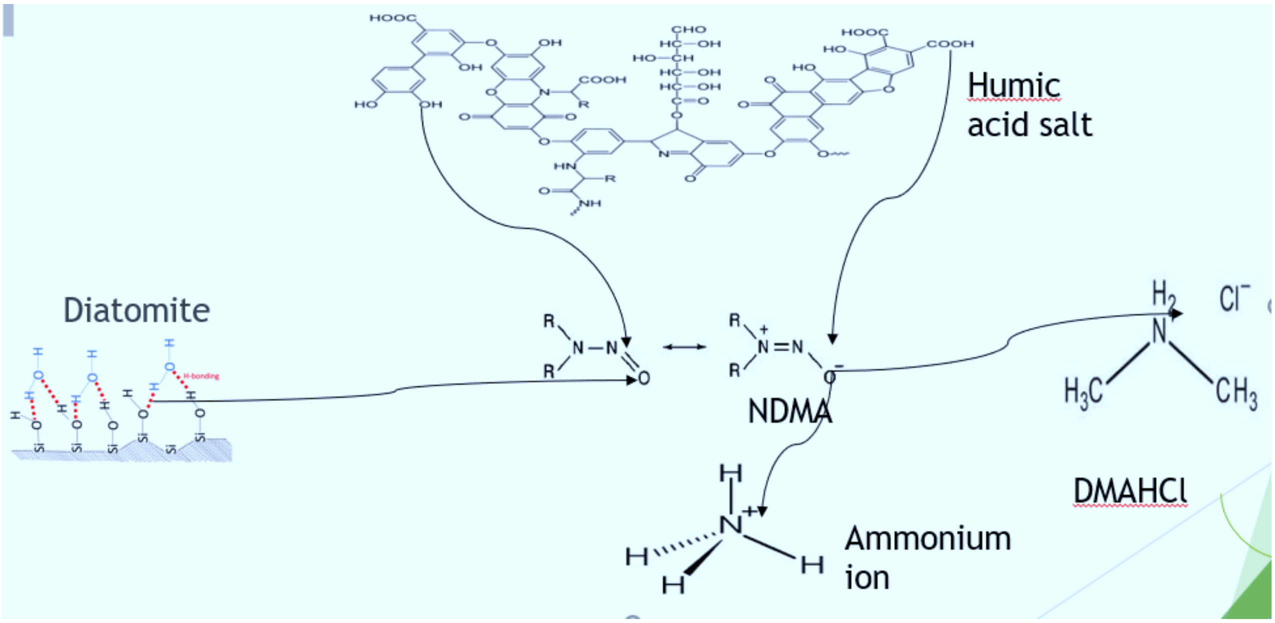

NDMA removal mechanism by rapid sand filtration

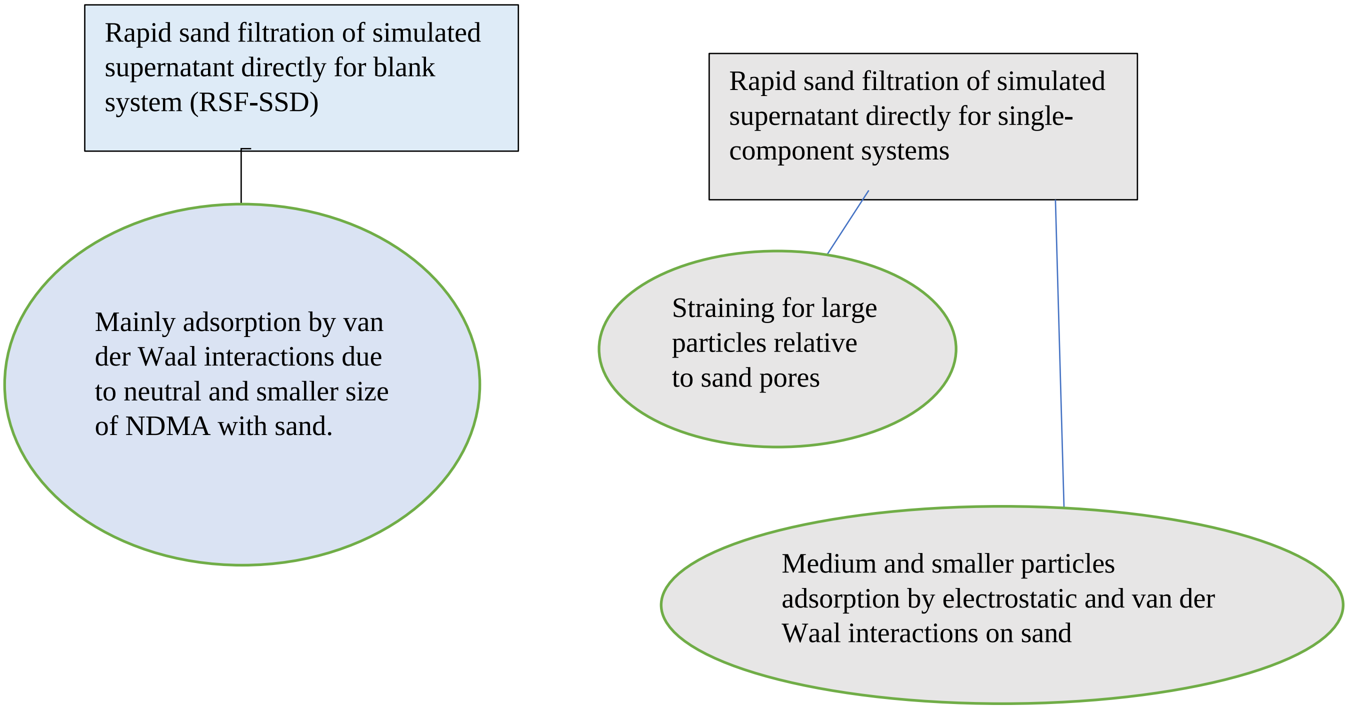

Figure S5: NDMA and water pollution parameters interactions, and NDMA removal mechanism by rapid sand filtration of simulated supernatant directly for blank and single-component systems.
